# High-Frequency Accessory and Transcapular Nerve Blocks in the Management of Fibromyalgia: A Case Report

**DOI:** 10.7759/cureus.76740

**Published:** 2025-01-01

**Authors:** Maimuna F Ahmed, Raveen K Aujla, Grigory Karmy

**Affiliations:** 1 General Medicine, Dr. Nandamuri Taraka Rama Rao (NTR) University of Health Sciences, Hyderabad, IND; 2 Interdisciplinary Medical Sciences (IMS), Western University, London, CAN; 3 Family Medicine, Faculty of Health Sciences, McMaster University, Hamilton, CAN

**Keywords:** accessory nerve block, case report, chronic pain mechanism, fibromyalgia, myofascial pain, pain management, peripheral nerve blocks, suprascapular nerve blocks, transcapular nerve block, weekly nerve blocks

## Abstract

This is the first case report describing the effect of weekly accessory nerve and transcapular nerve blocks for managing fibromyalgia (FM)-related pain in a 45-year-old female patient. The diagnosis was established using the new American College of Rheumatologist criteria. Following diagnosis, bilateral accessory and transcapular nerve blocks were administered using Xylocaine. In this particular patient, the nerve blocks decreased the numeric rating scale from 9 to 2 after treatment in the same visit, improved the benefits of daily activities, and improved tolerance of physical activities in six months. The patient reported significant improvement in pain, supporting the hypothesis that interventional management, like nerve blocks, may reduce peripheral nociceptive input and mitigate central sensitization, a hallmark of FM. The findings of this case report suggest that targeted nerve blocks can serve as a complementary treatment for FM-related neck and shoulder pain, particularly in cases involving myofascial trigger points in the trapezius and infraspinatus muscles. By integrating accessory and transcapular nerve blocks with existing multidisciplinary management approaches, clinicians can offer more options for pain management for FM patients with neck and shoulder pain. However, future randomized controlled trials are essential for cause-effect relationships and optimizing nerve block treatment protocols to support evidence-based practices and better patient outcomes.

## Introduction

Traditionally, fibromyalgia (FM) was considered a progression of myofascial pain syndrome (MPS). The hallmark of MPS is myofascial trigger points (MTrPs). MTrPs are found in muscles, ligaments, tendons, periosteum, scar tissue, or skin; they are characterized by a taut band within the muscle formed by uncontrolled contraction and shortening of the muscle and a twitch response that produces local and referred pain on palpation. The untreated MTrPs are the source of peripheral nociceptive input for central sensitization (CS), leading to the widespread pain characteristic of FM. However, FM is diagnosed by tender points, characterized by localized tenderness with an absence of referred pain, twitch response, and taut band, when, in fact, these tender points behave like MTrPs. Therefore, replacing the term tender point with trigger point may provide greater clinical and conceptual clarity in FM. In this case report, the term MTrPs is used instead of tender points [1].

Our concentration for this case report is on the trapezius and infraspinatus muscles, examining the administration of accessory and transcapular nerve blocks on a weekly basis in an adult female patient with FM. FM patients have active MTrPs in the trapezius, infraspinatus, and lower body muscles [2]. Patients with FM and migraine exhibited increased trapezius muscle stiffness [3]. A study found that the trapezius muscle pressure is almost three times higher in patients with FM, which may help explain the diffuse muscle pain of CS. Reducing this muscle pressure may change the clinical picture significantly by reducing the CS [4]. Spinal accessory and transcapular nerve blocks relax the MTrPs in the trapezius and infraspinatus muscles, respectively, relieving neck and shoulder pain commonly found in FM patients.

The gold standard for diagnosing FM is at the clinician's discretion. One of the 16 questionnaires proposed to evaluate FM is the new American College of Rheumatology (ACR) criteria, which includes questions related to associated symptoms of sleep disturbance, altered cognition, fatigue, irritable bowel syndrome, headaches, mood disorders, and various somatic symptoms [5]. This case report aims to demonstrate through a case presentation a hypothesis of the mechanism of action of nerve blocks in FM.

## Case presentation

A 45-year-old Canadian female primary school teacher initially presented with complaints of neck and shoulder pain immediately after a motor vehicle accident, which eventually turned into widespread pain within a month. In addition, she also reported sleep disturbance, mood disturbance, and cognitive dysfunction for eight months. Her past medical history includes migraine and hypothyroidism. The treatments tried in the past were muscle relaxants, diclofenac, OxyContin, amitriptyline, gabapentin, local lidocaine application, chiropractor treatments, physiotherapy, massage, and exercises, which gave minimal relief. FM was diagnosed using the new ACR FM criteria; the widespread pain index was 18/19, reflecting painful areas with MTrPs, and the symptom severity was 9/12.

In this case study, nerve blocks were integrated with standard therapies for the effective management of FM. Bilateral accessory nerve blocks were administered over the painful areas with 5 mL of 0.5% Xylocaine, a local anesthetic, on a weekly basis. Bilateral transcapular nerve blocks were administered over the painful areas with 7.5 mL of 0.5% Xylocaine weekly. Despite occasional flare-ups due to injury, traveling, stress, and increased activity, the patient consistently benefitted from the nerve blocks. Concurrent treatments included aquafit therapy, cognitive behavioral therapy (CBT), topical applications, and trigger point injections. Duloxetine was added eight months after nerve block treatment in response to stress-related pain triggers.

**Figure 1:**
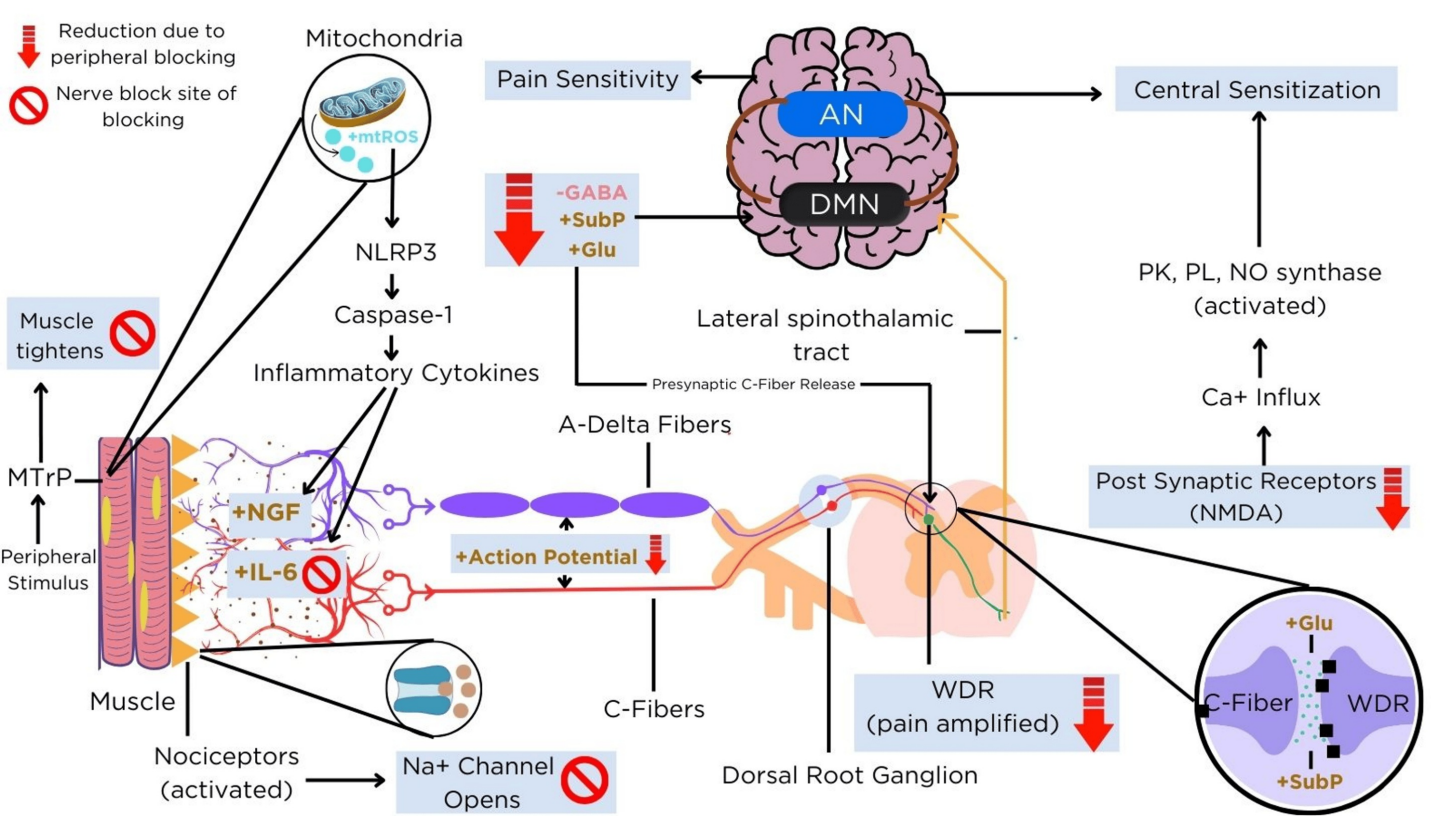
The mechanism of action of nerve blocks in the management of fibromyalgia A peripheral stimulus activates the MTrPs in the muscle, causing it to contract/tighten and altering muscle cell metabolism via the release of mitochondrial reactive oxygen species. Mitochondrial dysfunction within the muscle cells leads to the increased production of proinflammatory cytokines, such as nerve growth factor and Il-6, which interact with nociceptors within the muscle cells. The proinflammatory cytokines activate the nociceptors of the muscle cells, triggering the opening of sodium channels to depolarize the plasma membrane and generating an action potential. The action potential propagates through the A-delta fibers and C-fibers to the spinal cord’s dorsal horn, where they converge onto the WDR neurons. Neurotransmitters such as glutamate and substance P are released, activating the NMDA receptors on the postsynaptic neurons and driving CS. The postsynaptic signal then ascends the lateral spinothalamic tract to the brain’s anterior insula. Here, increased levels of excitatory neurotransmitters enhance cross-connectivity within the DMN and suppress coactivation of the AN, resulting in persistent pain and cognitive disruption. The prohibition symbols indicate the direct sites where the peripheral nerve blocks inhibit pain signaling. Red down arrows represent the reduction of variables in the pain pathway, including action potential propagation, neurotransmitter release, and NMDA activation, achieved through peripheral nerve block intervention AN: attentional network; DMN: default mode network; GABA: gamma-aminobutyric acid; Glu: glutamate; IL6: interleukin-6; mtROS: mitochondrial reactive oxygen species; MTrP: myofascial trigger point; NGF: nerve growth factor; NLRP3: nucleotide-binding domain, leucine-rich-containing family, pyrin domain-containing-3; NMDA: N-methyl-D-aspartate; NO: nitric oxide; PK: protein kinase; PL: phospholipases; subP: substance P; WDR: wide dynamic range The image was created using Canva.com

The patient reported pain relief lasting 7-10 days, leading to a better quality of life, improved capabilities in daily activities like housekeeping and childcare, and improved mental health. The patient returned to work and duties in six months. The numeric rating scale pain scores declined from 9 to 2 after treatment, with pain becoming less severe and more localized. No adverse events were reported. All sessions were tolerated well. The mechanism of action of nerve blocks in FM is shown in Figure 1.

## Discussion

Reception of pain and sensory pathway to the spinal cord

Pain signals originate at the peripheral nociceptors in the skin, muscle, tendon, or bursa [6]. MTrPs form the peripheral nociceptive input in FM [6]. The nociceptors of C-fibers (chronic pain sensory fibers) are polymodal. The nociceptors are stimulated by heat, pressure, vibration, injury, infection, stress, obesity, and depression in FM patients [7]. The nociceptors act as the transducers, converting the stimulus into action potential by opening the gates of the sodium channels and letting an influx of sodium, causing depolarization of the plasma membrane and creating the action potential [8]. The action potential travels through the A-delta fibers (myelinated) for sharp localized pain, and C-fibers (nonmyelinated) carry dull, diffuse, and burning pain associated with chronic pain. The small fiber neuropathy of C-fiber in 45% of FM shows increased spontaneous activity and sensitization to mechanical stimulation [9]. In addition, FM's neck and shoulder muscles have altered metabolism due to dysfunctional mitochondria, releasing inflammatory markers that interact with nociceptors [10]. Dysfunctional mitochondria release excessive levels of reactive oxygen species in the cytosol, activating inflammasomes such as nucleotide-binding domain, leucine-rich-containing family, pyrin domain-containing-3, and stimulating caspase-1, which increases several proinflammatory cytokines [10]. In particular, higher concentrations of inflammatory cytokines, such as interleukin (IL)-6, IL-8, IL-17, interferon-γ, and tumor necrosis factor-alpha (TNFα), were found in peripheral blood samples of FM patients [11].

Processing of pain in the dorsal horn of the spinal cord

The peripheral nociceptive impulses travel through the C- and A-fibers and converge into the wide dynamic range (WDR) neurons in the spinal cord's dorsal horn. The WDR neurons are the interneurons that help axons of afferent fibers synapse with other neurons in the pain pathway by either amplifying or inhibiting pain impulses. In CS, the WDR neurons amplify the pain by converting the touch sensation carried by A-beta fibers, close to noxious neurons, to noxious pain, called allodynia. The postsynaptic then ascends to the thalamus, hypothalamus, limbic system, and somatosensory cortex for sensory and affective aspects of pain. The activated C-fiber expresses excitatory neurotransmitters, glutamate, and substance P in the dorsal horn lamina 1 to 4 [7].

Pain amplification in CS

The postsynaptic neurons bear specific receptors. The neurokinin 1 receptors are activated by substance P, and N-methyl-D-aspartate (NMDA) and AMPA receptors are activated by glutamate. The excitatory neurotransmitter substance P and glutamate are elevated in FM, which amplifies the pain in CS [1].

Substance P diffuses further to expand the area of pain/receptive field, which is a character of CS. The release of substance P and glutamate in the synapse activates the NMDA receptors in the postsynaptic neurons. The activation of NMDA receptors is followed by calcium influx, activations of enzymes like protein kinase, phospholipases, and nitric oxide (NO) synthase, producing NO, as well as expression of c-fos, all of which contribute to a remarkable degree of CS. Neuroinflammation in the microglia of FM was shown in studies with translocator protein in the mitochondria and the release of cytokines. The progressive increase in pain in CS is due to the windup of second-order neurons producing dull or burning pain and unpleasant pain like throbbing or paresthesia that lasts longer than the stimuli, mediated through NMDA receptors [7]. CS can self-sustain without stimuli due to long-term neuroplasticity.

In the anterior insula of the brain, the increased concentration of glutamate, the excitatory neurotransmitter, relative to the inhibitory neurotransmitter (gamma-aminobutyric acid), enhances cross connectivity with the default mode network and reduces coactivation of the attentional network, which impairs the ability to shift focus from pain. Pain centralization alters brain network dynamics and causes increased pain sensitivity, persistent pain, and disrupted cognitive processes in FM [12].

Role of nerve blocks

CS can be dampened by intrinsic and extrinsic pain inhibition. Nerve blocks could act as extrinsic pain inhibitors. Nerve blocks administered weekly have reduced IL-6 in general chronic pain-like headache conditions, suggesting that similar markers could be reduced in FM patients [13]. FM patients receiving local anesthetic injections into the muscles had significantly reduced local hyperalgesia at the site of injection and hyperalgesia outside the site of injection, and pain decreased by 38% [14].

Local anesthetics in nerve block injections work by reversibly inhibiting sodium channels within the nociceptors at MTrPs, effectively deactivating peripheral stimuli and thereby blocking pain signaling. The inhibition of nociceptors diminishes action potential propagation in the C-fiber (slow, unmyelinated fibers) that transmit dull aching signals and Aβ-fibers that transmit touch as pain in chronic pain patients, reducing the transmission of pain signals from the peripheral input to the spinal cord by decreasing remote hyperalgesia [15,16]. Blocking the peripheral input causes the central processing to revert to normal [17]. An animal study provides insight into chronic pain management with local anesthetics. Nerve activity exhibits refractory, superexcitability, and depressive phases. In chronic pain, prolonged or repetitive nerve firing extends the depressive phase, rendering nerves more susceptible to blockade with lower doses of the anesthetic. Additionally, due to the low conduction safety in the nerve endings due to smaller nerve fibers, the nerve endings are more easily disrupted and less robust in maintaining electrical impulses when exposed to anesthetics [18]. Repeated administration of local anesthetic could ensure consistent and prolonged inhibition of nerve signal transmission, taking advantage of low conduction safety and maintaining effective pain relief by continuously disrupting the nerve signals.

Peripheral nerve blocks (PNBs) could have some advantages as a treatment option for chronic pain conditions characterized by CS, such as FM. PNBs have shown safety and efficacy in reducing pain intensity for migraines, which have a similar CS pathogenesis to FM [19]. Spinal accessory nerve blockade was effective in myofascial pain and was unresponsive to medications and physiotherapy. Unlike systemic medications like opioids, selective serotonin reuptake inhibitors (SSRI), and serotonin and norepinephrine reuptake inhibitors (SNRI), PNB minimizes central nervous side effects such as sedation, confusion, and the addictive potential of opioids. PNB could reduce negative psychosocial consequences associated with FM, like unemployment and long-term disability payments, by controlling pain without contributing to the opioid crisis. By alleviating pain, PNBs motivate participation in physical exercise programs, an essential part of FM management that typically turns into a challenge due to pain. A single nerve block can cover multiple muscles, reducing widespread intervention. While medications can lose effectiveness over time, PNB can lower ectopic pain impulse generation and propagation, providing consistent pain relief.

The 2012 Canadian guidelines for FM management include exercise therapy (aerobic exercises, pilates, tai chi, yoga, and Tui Na), psychological therapy (traditional CBT, group therapy, and motivational interviewing), analgesics, opioids, cannabinoids, antidepressants (tricyclic antidepressants, SSRIs, and SNRIs), anticonvulsants (gabapentinoids), dopaminergic agents, and sleep modifiers [20]. PNBs could complement these by reducing pain and enhancing the effectiveness of other treatments.

Strengths and limitations

The strength of this case report is the generation of hypotheses that nerve blocks could be effective in FM through its unique mechanism of action at various levels of the pain pathway. The clinical efficacy of nerve blocks for FM-related pain management could be tested with formal research methods through comparative studies that are observational or randomized controlled trials. This case report also carries educational value in understanding the basic science behind nerve blocks in FM, contributing to medical knowledge, and enhancing communication between clinical and academic fields. The cost of conducting this report is low as the cases are taken from regular clinical settings, which has the potential to increase options for pain management in FM.

The limitation is that the findings from this case report cannot be generalized due to a lack of representative population and sample size. This case report was analyzed retrospectively with no control group to compare, giving a lower level of evidence. The outcomes were measured using the patient’s experience, which can be subjective and variable.

## Conclusions

The case report suggests that accessory and transcapular nerve blocks are promising tools in the multidisciplinary management of FM. Their targeted approach addresses both peripheral and central components of pain, complementing standard therapies. FM is primarily considered a CS condition, though peripheral nociceptive inputs like the MTrPs influence it. MTrPs are a better conceptual and clinical replacement for tender points in FM diagnosis. Peripheral nociceptive input, particularly from trapezius and infraspinatus, contributes to CS and widespread pain. Accessory and transcapular nerve blocks can effectively reduce peripheral pain inputs by relaxing the MTrPs in the muscles, blocking the sodium channel, and reducing nociceptive signaling. Nerve blocks have fewer side effects than systemic medications, which may reduce dependence on opioids. The unique pathogenesis of FM includes altered trapezius muscle activity, elevated trapezius muscle pressure, mitochondrial dysfunction, neuroinflammatory processes, and pain amplification. Integrating peripheral interventions like nerve blocks with standard therapies could provide effective management for FM, addressing both peripheral and central components. Future randomized controlled trials are needed to establish cause-effect relationships and optimal treatment protocols, including frequency, duration, and concentration of local anesthetics in the nerve blocks. These will support evidence-based practices, refine treatment strategies, and improve outcomes using validated assessment tools. This case report is published with written informed consent of the patients.
